# Reflections on a Health System's Telemedicine Marathon

**DOI:** 10.1089/tmr.2020.0009

**Published:** 2020-11-18

**Authors:** Lawrence R. Wechsler, Srinath Adusumalli, Mary Elisabeth Deleener, Ann Marie Huffenberger, Gregory Kruse, C. William Hanson

**Affiliations:** ^1^ Department of Neurology, Perelman School of Medicine, University of Pennsylvania, Philadelphia, Pennsylvania, USA.; ^2^ Department of Cardiology, Perelman School of Medicine, University of Pennsylvania, Philadelphia, Pennsylvania, USA.; ^6^ Department of Anesthesiology, Perelman School of Medicine, University of Pennsylvania, Philadelphia, Pennsylvania, USA.; ^3^ Office of the Chief Medical Information Officer, University of Pennsylvania, Philadelphia, Pennsylvania, USA.; ^4^ Penn Medicine Center for Connected Care, University of Pennsylvania, Philadelphia, Pennsylvania, USA.; ^5^ Perelman School of Medicine, University of Pennsylvania, Philadelphia, Pennsylvania, USA.

**Keywords:** telemedicine, telehealth, health system

## Abstract

The coronavirus disease 2019 (COVID-19) public health emergency necessitated changes in health care delivery that will have lasting implications. The University of Pennsylvania Health System is a large multihospital system with an academic medical center at its core. To continue to care for patients with and without COVID-19, the system had to rapidly deploy telemedicine. We describe the challenges faced with the existing telemedicine infrastructures, the central mechanisms created to facilitate the necessary conversions, and the workflow changes instituted to support both inpatient and outpatient activities for thousands of providers, many of whom had little or no experience with telemedicine. We also discuss innovations that occurred as a result of this transition and the future of telemedicine at our institution.

## Introduction

Similar to many large health systems in the years before the coronavirus disease 2019 (COVID-19) pandemic, the University of Pennsylvania Health System (UPHS) devoted increasing resources and attention to connected health with goals of systematically enhancing patient outcomes, expanding patient access, and facilitating community engagement. Before the pandemic, the PENN E-LERT^®^ program provided remote monitoring of >250 intensive care unit (ICU) beds. Inpatient and ambulatory virtual visits were performed by many primary and specialty services using video capabilities of the health systems' electronic medical record (EMR; Epic Systems, Inc., Verona, WI). Penn Medicine OnDemand (PMOD) provided urgent virtual care services also utilizing the EMR. The UPHS was an early adopter of remote patient monitoring after discharge from the hospital to manage chronic conditions such as congestive heart failure and chronic obstructive pulmonary disease. All connected health activities at UPHS were coordinated through an enterprise Office of the Chief Medical Information Officer. The UPHS administratively partitioned connected health activities that operate a centralized care delivery model applicable across the health system (e.g., Penn E-Lert, PMOD) from network telemedicine activities (e.g., legal, regulatory, reimbursement, and technology) that service department-specific telemedicine applications.

**FIG. 1.**
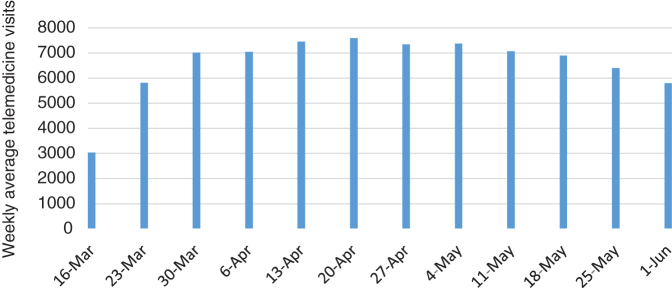
Weekly average daily telemedicine visits from the start of the pandemic in March through June.

Much of this changed in mid-March 2020 due to the public health emergency and policy waivers instituted to facilitate the adoption of and conversion to telehealth.^1^ Outpatient facilities were closed to avoid transmission of infection. Hospital procedures were altered to minimize contact with COVID-19 and preserve personal protective equipment (PPE). Telemedicine offered an optimal path to continue care for patients concerned about potential infection from exposure at health care facilities. At UPHS, similar to other large health systems,^2,3^ the volume of telemedicine encounters within weeks increased from <100 per day to >7000 daily encounters (Fig. 1). This new reality required realignment of technology, organization, communication, and operations, transforming the UPHS telemedicine capability by the end of March to the dominant mode of care delivery throughout the organization (Table 1).

**Table 1.** Changes in University of Pennsylvania Health System Telemedicine Functions to Address Infection Control Before and During the COVID-19 Pandemic

	Prepandemic	During pandemic
Outpatient virtual visits	Telemedicine used for outpatient visits in limited role with <100 visits per day. Videoconferencing using on-premise server-based technology with limited capacity for expansion.	Conversion of most outpatient visits to telemedicine to avoid potential exposures in hospital clinic environment. Videoconferencing changed to cloud-based platform. Support through command center.
Inpatient teleconsultations	Inpatient teleconsults in selected areas including telestroke and critical care.	Deployment of iPads on stands throughout Penn hospitals to allow providers and staff to reduce exposures and conserve personal protective equipment. Access to all devices including patient's personal phones from proprietary Penn Medicine Switchboard application developed at University of Pennsylvania Health System.
Penn Medicine OnDemand	Service available primarily to faculty, staff, and students through MyChart portal.	Expanded availability by increasing staffing to accommodate dramatic increase in volume and to limit need for in-person evaluations at emergency departments, urgent care, or outpatient offices. Continued to utilize MyChart portal.
Penn Medicine at Home	Monitoring of selected patients at home with visits by home health care staff.	Applied home monitoring to COVID-19 patients with mild-to-moderate disease capable of management at home with remote monitoring to maintain availability of hospital beds for more severe patients with respiratory distress.

## Scaling Telemedicine During the Pandemic

When expansion to thousands of encounters daily became necessary within a matter of days, the existing legacy audio/video platform did not prove scalable. The integrated solution requiring providers to work on secure but highly customized UPHS telemedicine workstations was impractical with competing demands for hardware due to significant supply chain disruptions. The UPHS quickly adopted a cloud-based scalable videoconferencing platform that could accommodate the increase in telemedicine activity across all settings. Initially, the majority of patient visits were performed by telephone but implementation of this scalable platform allowed the health system to gradually convert to telemedicine visits, ultimately peaking by early April at 70% of all patient encounters. The conversion from telephone to audiovisual telemedicine increased provider and patient satisfaction and improved reimbursement that in the early stage of the pandemic was significantly higher for video visits. Telemedicine encounters continued at high levels throughout the pandemic until August when the system began to gradually restart limited in-person visits. Our experience was similar to reports from other health systems throughout the country.^3,4^

## Centralizing Essential Telemedicine Functions for the System

To serve many providers using telemedicine for the first time as well as a new platform and workflow, a technical support command center was created to respond to all technology-related issues. In addition, there was a prominent online command center presence with access both from within the system and through a remote access portal. Telemedicine-related material was posted to an UPHS website including tip sheets for utilizing the telemedicine platform, video microlearnings, aids for connecting with patients, and information regarding legal, regulatory, billing, and licensing aspects of telemedicine. The command center was staffed by information system personnel knowledgeable about telemedicine. The command center was available by phone for technical and workflow-related issues experienced by providers performing outpatient telemedicine visits including setup, scheduling, and audio or video difficulties. Similarly, connectivity and equipment problems arising during inpatient telemedicine visits were addressed when necessary by the command center. Administrative and clinical leaders in telemedicine were identified within departments and divisions and empowered to transmit important items to their colleagues through departmental meetings and e-mail communications. There were daily and then weekly teleconferences for these champions with telemedicine leadership to provide updates and clarifications. The command center remained functional during the period of high-volume telemedicine activity and transitioned support to individual departments as in-person outpatient and inpatient visits returned.

The UPHS also centralized policies regarding reimbursement and coding. Reimbursement policies for video and telephone visits changed frequently during the pandemic. The health system decided the best approach would be for providers to follow their usual practices, indicate whether the visit was telephone or audio/video telemedicine, and defer management of billing and coding to centralized backend billing and coding specialists armed with up-to-the minute regulatory updates. A telemedicine documentation template was created that was convenient for providers but also captured elements (in structured form) known and predicted to be necessary for billing. Appropriate modifiers were available selections when choosing a billing level. Other templates outlined telephone or video-based standard examinations that encompassed the essential aspects accessible in a telemedicine visit. These documentation aids maximized reimbursement opportunities and powered a system-wide analytics dashboard.

## Inpatient Telemedicine

Early in the pandemic, PPE such as masks, gowns, and face shields was in short supply. In addition, every person entering rooms of patients with COVID-19 or with uncertain COVID-19 status faced potential exposure. Telemedicine allowed care to be delivered while minimizing risks to providers and patients. In non-ICU rooms without existing cameras, iPads were set up on stands to permit direct communication between providers and patients. Telemedicine was used by primary teams on rounds to limit exposure in most cases to one person on the team while others participated remotely. Specialists performed consultations through the iPads unless it was thought that an in-person examination was necessary. Nurses and other clinicians could also respond to patients for nonurgent matters without donning PPE and physically entering the room. Because of the prohibition against visitors, a critical function of the iPads was family communication with patients to reduce isolation and anxiety. Videoconferencing was frequently applied to care team meetings with family members permitting participation from locations well beyond the local area.

A similar concern arose in the emergency department (ED) where worried family members wanted to keep apprised of the status of their loved ones. Similar to the patient floors, iPads in the ED were used for consultations whenever both the providers and ED staff considered a telemedicine visit adequate. As the technological advances progressed, patients in the ED and on the floors could register their own cellular phones with UPHS, permitting providers and staff to connect directly with patients using the UPHS videoconferencing platform even if an iPad was not available. Screening tents for patients with possible COVID-19 symptoms were also equipped with iPads enabling remote evaluations, further reducing exposures and conserving PPE.

## Outpatient Telemedicine

In outpatient practices, encounters were converted to telemedicine to maintain access to providers and support existing patients by limiting contacts and potential exposure to COVID-19. In most practices, clinic staff or support personnel from activities on hold during the pandemic were redeployed to contact patients ahead of their visits, walk them through necessary steps for videoconferencing communications, and test the audio and video capabilities of their device. This proactive process helped assure a successful video visit at the time of the appointment without the need to struggle with technology issues. Visit reminders containing telemedicine appointment and setup information were automatically sent to patients at the time the appointment was made and then again 48 h before the visit.

**FIG. 2.**
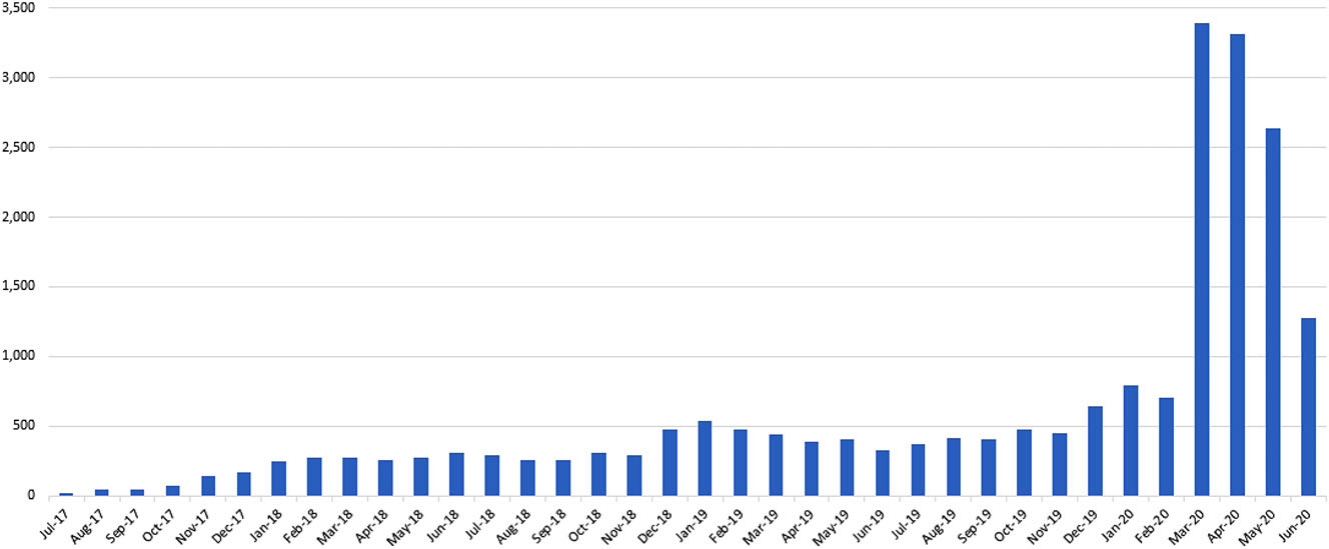
Penn OnDemand telemedicine encounters (by month).

Virtual care visits through PMOD before the COVID-19 crisis mostly serviced the Penn community including staff, faculty, students, and patients registered on the Penn portal (myPennMedicine). With concerns about symptoms due to COVID-19, the UPHS extended availability of PMOD, resulting in a nearly 500% increase in calls and requiring a rapid escalation in staffing and availability (Fig. 2). Providers reassured those without typical COVID-19 symptoms and referred those with possible infections for testing. For severe breathing difficulties, patients were referred to emergency rooms. The availability of this virtual care practice significantly reduced the burden on emergency rooms and urgent care locations and reduced exposure to potentially infectious patients.

Penn Medicine at Home was another pre-existing service that assumed new functions during the COVID-19 crisis. Patients with moderate symptoms of COVID-19 without danger of immediate respiratory compromise could be safely monitored at home. Remote patient monitoring technology promoted frequent communication, and home nurses advised patients to return if they deteriorated. The health system benefited from integration of the home care service with providers across the entire continuum of care on a single EMR enabling earlier discharge for stable patients.

## Telemedicine Innovations During COVID-19

The dramatic expansion of telemedicine resulted in several innovations in response to requests from providers to address logistical problems. Only a few dedicated telemedicine carts were operationalized in the health system at the start of the COVID-19 crisis, and a solution was needed to supply increased videoconferencing capabilities to multiple hospitals. COVID-19 patients quickly overwhelmed the existing ICU beds requiring repurposing of non-ICUs to accommodate acutely ill patients including many on ventilators. Telemedicine connections using iPads positioned in patient rooms or moved from room-to-room allowed critical care intensivist support from the Penn E-lert command center to supplement providers recruited to work in ICUs. Over 800 iPads were procured within a few days to meet the urgent demand for connected health. Penn E-lert intensivists were able to consult with the ICU clinicians, recommend actions to treat patient conditions, and support high-quality care.

A significant enhancement to the outpatient experience was achieved through the development of the Penn Virtual Visit Switchboard. The switchboard could be used for both outpatient telemedicine visits and inpatient telemedicine consults. When a provider logged into the switchboard, they first chose outpatient or inpatient functions bringing them to the appropriate site. To facilitate outpatient visits, a provider's patient schedule was imported through the EMR into the switchboard, allowing one-click access to the virtual examination room corresponding to each patient encounter. The switchboard indicated whether the patient was already connected to the room and allowed providers (or their staff) to send text and e-mail messages such as “I am running late and will be with you shortly,” directly to the patient. If technology checks were completed before the visit, a note was included on the switchboard. A chatbot was added to guide patients in setting up telemedicine encounters. When inpatient functions were chosen, the switchboard displayed patient lists for each unit and hospital in the enterprise. For each patient, the switchboard listed available devices for connection to patients such as personal phone, dedicated iPad or shared iPad, and a link to connect to each device through a secure meeting room. The switchboard automated tasks that previously required staff time and greatly simplified the process of connecting to both inpatients and outpatients.

## Lessons Learned

Telemedicine has strengths and weaknesses and both were apparent during this pandemic. It is clear that we need to know more about the efficacy of telemedicine for various patient phenotypes, situations, diagnoses, and specialties. Further research is needed comparing telemedicine with in-person visits examining not only satisfaction but also clinical outcomes, accuracy of diagnosis, and equity of care. The tremendous increase in connected health activity during the pandemic opened many opportunities to evaluate telemedicine and its utility under a variety of circumstances. Studies at UPHS are ongoing and the outcomes will help clarify the optimal role of telemedicine among the options for medical care once in-person visits are again available.

## UPHS Telemedicine after COVID

Telemedicine evolved slowly over the past decade due to technology, reimbursement, and regulatory restrictions. The COVID-19 public health emergency clearly demonstrated the potential for telemedicine to become a primary modality for providing care to selected patients. Although many current telemedicine regulations and waivers are temporary or expire when the COVID-19 public health emergency ends, it is likely that some of the changes will survive. Given the satisfaction with telemedicine expressed by both providers and patients, Centers for Medicare & Medicaid Services and private insurers should recognize the benefits of telemedicine and respond favorably to the desires of their members. Telemedicine will become an integral component of care delivery and assume a complementary role to in-person medicine. For patients with reduced mobility or with limited access to an UPHS facility, telemedicine may become the standard and the health system must address the digital divide restricting access in some areas and populations. With barriers removed and payer support of telemedicine, the provider community should determine the optimal combination of modalities to deliver high-quality, cost–effective, and patient-centered care. The experience of centers such as the UPHS during the pandemic helps advance the ability to deliver telemedicine services across a broad spectrum of services, providers, and patients.

## Abbreviations Used

COVID-19: coronavirus disease 2019
ED: emergency department
EMR: electronic medical record
ICU: intensive care unit
PMOD: Penn Medicine OnDemand
PPE: personal protective equipment
UPHS: University of Pennsylvania Health System

## References

[1] Moore MA, Munroe DD. COVID-19 brings about rapid changes in the telehealth landscape. Telemed J E Health 2020. [Epub ahead of print]; DOI: 10.1089/tmj.2020.0028. 32804048

[2] Meyer BC, Friedman LS, Payne K, et al. Medical undistancing through telemedicine: a model enabling rapid telemedicine deployment in an academic health center during the COVID-19 pandemic. Telemed J E Health 2020. [Epub ahead of print]; DOI: 10.1089/tmj.2020.0327. 33030985

[3] Demaerschalk BM, Blegen RN, Ommen SR. Scalability of telemedicine services in a large integrated multispecialty health care system during COVID-19. Telemed J E Health 2020. [Epub ahead of print]; DOI:10.1089/tmj.2020.0290. 32795147

[4] Grossman SN, Han SC, Balcer LJ, et al. Rapid implementation of virtual neurology in response to the COVID-19 pandemic. Neurology 2020;94:1077–1087. 32358217 10.1212/WNL.0000000000009677

